# Advanced Functional Materials for Electrochemical and Biosensors

**DOI:** 10.3390/bios14050248

**Published:** 2024-05-15

**Authors:** Khursheed Ahmad

**Affiliations:** School of Materials Science and Engineering, Yeungnam University, Gyeongsan 38541, Republic of Korea; khursheed@yu.ac.kr

## 1. Introduction

Modern science and technology are central to the smooth running of daily life [1]. The modern world relies on gadgets and appliances such as mobile phones, computers, microwave ovens, air conditioners, televisions with remote controls, smoke detectors, LED lights, fans, and infrared (IR) thermometers, which provide support and enable us to interact with the physical environment [2]. Most of these items employ sensors, which are devices which can detect changes in chemical, physical, or biological conditions and convert these changes into signals that can be recorded or measured. Sensors are used in a wide range of applications and industrial process control, including environmental monitoring, medical diagnostics, and consumer electronics [3], and can also detect various parameters, such as motion, temperature, humidity, pressure, light, and chemical substances [4]. They play a crucial role in modern technology by providing valuable information for decision-making and automation. Various kinds of sensors have been developed, such as optical, electrochemical, fluorescence, temperature, motion, proximity, gas, light, biometric, humidity, and chemical sensors [5]. Temperature sensors are generally used in weather stations, thermostats, and industrial processes; proximity sensors are used in smartphones, automation, and robotics; motion sensors are used in gaming consoles, security systems, and automatic doors [6]; pressure sensors are utilized in medical and automotive applications; light sensors are widely used in automatic lighting systems, cameras, and solar panels [7]; gas sensors can be used to monitor the presence of gases in the environment and monitor air quality; biometric sensors are next-generation sensors used in healthcare devices, security arms, and smartphones [8]; and chemical sensors are used to detect chemicals and their properties for the monitoring of various biomolecules or toxic compounds in the environment and healthcare systems. Electrochemical sensors have received much attention because of their excellent sensitivity, selectivity, reproducibility, stability, and detection limits; ease of fabrication; simple processing; cost-effectiveness; portability; and repeatability compared to conventional sensing techniques [9]. Similarly, biosensors are electro-analytical devices that employ a biological component with a physicochemical detector to detect the presence of a specific substance or analyte. Electrochemical sensors and biosensors almost work using the same principle, except the difference in their electrode materials. Sensors are generally fabricated using nanostructured or polymeric materials and used for the determination of chemicals such as hydrazine, hydrogen peroxide, catechol, etc. [10]. In the case of biosensors, nanostructured electrode materials with biological enzymes such as glucose oxidase are used for the fabrication of biosensors, which are used in the determination of biomolecules such as dopamine, glucose, ascorbic acid, uric acid, etc. [11]. Sensors and biosensors are widely used in various fields, such as food safety, medical diagnostics, and environmental monitoring. Sensors and biosensors record signals to determine the electrochemical oxidation or reduction of analytes. The detection behavior of sensors and biosensors relies on the physiochemical properties of the electrode materials.

Advanced functional materials such reduced graphene oxide, metal oxides and polyaniline (PANI), and carbon nanotubes (CNTs) and their composite materials possess excellent electro-catalytic, electrical, thermal, and conductive properties [12]. Advanced functional materials have several advantages compared to traditional materials for sensing and biosensing applications. In the past few decades, various advanced functional materials have been widely used in the development of sensors and biosensors. This Special Issue involves the fabrication of sensors and biosensors using advanced functional materials and contains ten articles and two communications, which are briefly elaborated upon in the next section. The aim of this editorial is to encourage readers to explore these published articles and communications. This Special Issue may be useful for the scientific community working on the design and preparation of advanced functional materials towards the development of sensors and biosensors.

## 2. Summary of the Contributed Articles

In this Special Issue, 15 articles were submitted, but 12 articles (10 research articles and 2 communications) were published. In this section, an overview and summary of the published articles are provided. In the first study, Chaudhary et al. [1] synthesized cerium oxide (CeO_2_) nanoparticles (NPs) using cerium nitrate as the precursor via a simple precipitation method. The authors used a calcination process to obtain the CeO_2_ NPs. The phase and crystalline nature of the obtained CeO_2_ NPs were characterized by X-ray diffraction (XRD), whereas scanning electron microscopy (SEM) was employed to assess the surface morphological features. The XRD method showed the good crystalline nature of the prepared CeO_2_ NPs, while the SEM results indicated that CeO_2_ NPs had particles sizes of less than 100 nm, and that the particles were agglomerated. Energy-dispersive X-ray (EDX) spectroscopy and X-ray photoelectron spectroscopy (XPS) approaches showed the formation of CeO_2_ NPs with good phase purity. Furthermore, the authors fabricated a glassy carbon electrode (GCE) using CeO_2_ NPs as the electrode material. The electrochemical properties of the fabricated electrode (CeO_2_/GCE) were investigated in the presence of a 5 mM [Fe(CN)_6_]^3−/4−^ redox system using electrochemical impedance spectroscopy (EIS) and cyclic voltammetry (CV). The authors found that CeO_2_/GCE has good electro-catalytic properties and can be applied as the working electrode for the determination of hydroquinone (HQ). Firstly, the authors used CV to detect the HQ and the effects of different scan rates, and the obtained results demonstrated that the sensing of HQ on the CeO_2_/GCE surface is an adsorption-controlled process. The authors also assessed the effects of various concentrations on the performance of CeO_2_/GCE using CV and found that the current response of redox reactions increases with respect to the concentration of HQ. The authors also proposed that differential pulse voltammetry (DPV) may be a more sensitive technique and recorded DPV graphs of CeO_2_/GCE in the presence and absence of HQ. The obtained results showed that CeO_2_/GCE has higher performance compared to the bare GCE. The obtained results showed good sensitivity of 0.41 µA/µM.cm^2^ and a detection limit of 0.9 µM. It was also reported that CeO_2_/GCE has excellent selectivity for the determination of HQ in the presence of glucose, H_2_O_2_, urea, dopamine, uric acid, acetone, chlorophenol, hydrazine, and ascorbic acid. CeO_2_/GCE also exhibited excellent repeatability and stability.

Quasim et al. [2] examined the sensing properties of hydrothermally grown manganese dioxide (α-MnO_2_) for the development of a serotonin sensor. Serotonin is a well-known monoamine neurotransmitter which is generally considered to play an important part in physiological and biological processes. Thus, the authors developed a serotonin sensor using α-MnO_2_ as a sensing material, and GCE was coated with α-MnO_2_ and CV. The results showed that α-MnO_2_-modified GCE has better performance than bare GCE. The CV and DPV techniques were used to conduct examinations and perform sensing experiments in the presence of serotonin. The authors found that the current response increases with increasing concentrations of serotonin, and it was also further reported that the sensing process is adsorption-controlled rather than diffusion-controlled. The α-MnO_2_-modified GCE demonstrated good cyclic stability of 50 cycles, and selectivity for serotonin detection in the presence of various interfering compounds. The authors also reported a detection limit of 0.14 µM with sensitivity of 2.41 µA/µM.cm^2^. The fabricated electrode also demonstrated excellent storage stability and repeatability, which suggested its potential for further practical applications.

G. Silva-Galindo and M. Zapata-Torres [3] reported the fabrication of a non-enzymatic glucose biosensor. The authors fabricated an anatase phase of titanium/titanium dioxide (Ti/TiO_2_) NPs using a simple spin-coating method via a polymeric precursor approach. It was observed that Ti/TiO_2_ has an irregular surface with clusters of NPs. The electrochemical characterization of Ti/TiO_2_ was performed using CV for the detection of glucose. This fabricated biosensor showed interesting electrochemical performance in terms of the detection limit and sensitivity. The authors also conducted amperometric investigations for the detection of glucose, and a wide linear range was also observed. A real sample analysis was also carried out in sweat and saliva samples, which showed decent real sample practical applicability of the fabricated glucose biosensor.

Mollamohammadi et al. [4] studied the electrochemical sensing properties of carboxymethyl starch graft PANI/multi-walled carbon nanotube composites (CMS-g-PANI@MWCNTs). The authors further modified GCE using CMS-g-PANI@MWCNTs as an electro-catalyst. Furthermore, tyrosinase was immobilized to fabricate L-dopa and catechol biosensors. The fabricated CMS-g-PANI@MWCNTs/Tyrase/GCE-based biosensor exhibited linear ranges of 5–100 and 10–300 µM with sensitivity of 2.4 and 1.11 µA/µM.cm^2^ and detection limits of 25 and 30 µM, for catechol and L-dopa, respectively. The authors proposed that the CMS-g-PANI@MWCNTs/Tyrase/GCE biosensor may be used for practical applications due to its excellent performance in real samples.

Demkiv et al. [5] reported that L-lactate is used in food quality determination, and thus, developed an L-lactate biosensor using novel strategies. In this study, flavocytochrome b_2_ (Fcb_2_) was used as a bio-recognition element, whereas electroactive platinum and gold NPs were adopted for enzyme immobilization for the fabrication of the L-lactate biosensor. The authors isolated the enzyme from yeast using thermotolerant yeast Ogataea polymorpha. This fabricated biosensor demonstrated a good detection limit of 0.010 mM and a linear range up to 0.12 mM. The authors also examined storage stability and found that the fabricated L-lactate sensor has a storage stability of 7 days. The proposed biosensors fabricated via Fcb_2_-mediated electro-active NPs displays great promise for use in food control laboratories.

Wang et al. [6] examined the electrochemical sensing properties of a Au/electropolymerized polyaniline–polystyrene sulfonate (PANI: PSS)-modified scree-printed carbon electrode (SPE). This fabricated electrode was further immobilized with urease and was explored for the determination of urine samples. Urine analysis is a widely used medical test in healthcare systems to indicate human health status, and is often used to diagnose chronic kidney disease. In urine tests, ammonium ions, urea, and creatinine are the main clinical indicators in urine tests for chronic kidney disease. The authors reported the CV method for the determination of urine samples. AuNP/PANI: PSS/SPE was used for the determination of NH_4_^+^ ions, and they reported a detection limit of 290.1 µM with sensitivity of 192.6 mA/M.cm^2^ and a linear range of 0.5–20 mM. Further, the authors used AuNP/PANI:PSS/urease/anion-exchange membrane/SPE for the sensing of urea and reported a detection limit of 500 µM with sensitivity of 106.8 mA/M.cm^2^ and a linear range of 0.5–15 mM. Subsequently, AuNP/PANI:PSS/creatinine deiminase/chitosan/SPE was used as creatinine sensor, which exhibited a detection limit of 562.5 µM and sensitivity of 62.34 mA/M.cm^2^. The authors proposed that this device can be further explored for the determination of other analytes, such as albumin and glucose, in the future.

Saxena et al. [7] studied the electro-catalytic behavior of rGO/Au-modified MIP on SPE using EIS and CV techniques. The CagA antigen plays a crucial role as the primary virulence factor in initiating *H. pylori* infection in the human stomach. Therefore, the suggested MIP-based biosensor holds potential for future development as an early-stage gastric cancer detection tool for use at the point of care. In this regard, the authors prepared an electrode material (rGO/AuNPs) using electro-deposition method, and the fabricated electrode was utilized to determine the CagA antigen of *H. pylori*. ESP and frontier orbital analysis were used to determine the reactive sites of functional monomers and the CagA template, revealing potential atomic contacts that play a key role in the formation of imprinted sites. The effects of temperature and pH were also optimized, with a detection limit of 0.05 ng/mL and sensitivity of 0.275 µA/ng.mL. Therefore, the proposed biosensor using MIPs could potentially be developed into a point-of-care device for the detection of early-stage gastric cancer.

Elfarargy et al. [8] conducted an experiment supported by DFT calculations to determine electrochemical sensing properties of a graphitic carbon nitride sheet (g-CN)-based electrode. Methotrexate (MTX) was used as the sensing analyte, and square-wave voltammetry (SWV) and CV were utilized for the determination of MTX. The authors modified the carbon paste electrode (CPE) with g-CN as the electrode modifier and used it for the detection of MTX in various concentrations, reporting a detection limit of 12.45 nM at pH 7.0. The effect of the applied scan rate suggested that the sensing process is diffusion-controlled, and further investigations involving real sample analysis (urine sample, plasma and table samples) suggested its potential for practical applications.

Tamiya et al. [9] focused on the development of the rapid detection of anti-SARS-CoV-2-neutralizing IgG/slgA antibodies and antioxidant activity in saliva. The authors fabricated a biosensor which exhibits dual-functional properties enabling the determination of both antibodies against SARS-CoV-2 and the measurement of anti-oxidant activity. Nineteen saliva and serum samples were gathered over a ten-month period, with collection occurring three weeks following the initial vaccine, eight months after the second vaccine, and one month following the third vaccine. After vaccination, the antibody concentrations varied within the following ranges: serum IgG: 81–15,000 U/mL, salivary IgG: 3.4–330 U/mL, and salivary IgA: 58–870 ng/mL. A significant rise in salivary IgG levels occurred following the second vaccination. The levels of sIgA also displayed a rising pattern, which correlated with the trends seen in serum IgG levels. This suggests the potential for saliva as a regular means of evaluating vaccine effectiveness. The electrochemical immune-sensor assay fabricated in this study, using a Au-linked electrochemical immunoassay, and the measurement of antioxidant activity based on luminol electrochemiluminescence (ECL) can be conducted with portable devices. This would be beneficial for individualized diagnosis using saliva samples. The development of these biosensors could have significant implications for public health, as they offer a convenient and non-invasive method for monitoring immunity and health status in individuals, particularly in the context of the COVID-19 pandemic.

Murray et al. [10] developed a five-stranded four-way-junction multi-purpose biosensor for the detection of genetic signatures of the SARS-CoV2 (genes N and S) and influenza A (gene M) viruses. The fabricated biosensor involves the use of two universal strands (UMeB and USL) and two target specific strands (m and f) which can be simply tuned for the determination of different pathogens. The 5S-4WJ configuration comprises an electrode-5 universal stem–loop (USL) strand, two additional DNA strands, and a universal methylene blue redox strand (UMeB). The use of SPE and a versatile design for certain biosensor parts enables the fabrication of low-cost electrochemical biosensors. Furthermore, integrating this biosensing platform with NASBA amplification offers a sensitive technique suitable for real-world applications.

Sanyal et al. [11] reported density functional theory calculations for the investigation of the electronic, structural, and sensing properties of pristine and defected VSe_2_ monolayers. The authors investigated the stability of VSe_2_(Se_v_) + NH_3_ and VSe_2_(V_v_) + NH_3_ monolayers by examining their adsorption energy values. VSe_2_(Se_v_) exhibited stronger binding with the adsorbed NH_3_ molecule compared to the pure nanomaterial. The authors also found that introducing a Se vacancy causes the adsorption energy to increase from −0.12 eV in the pristine case to −0.97 eV for VSe_2_(Se_v_). Moreover, they reported that the stronger adsorption of NH_3_ on defected VSe_2_ may be attributed to charge transfer, where NH_3_ acts as a donor and VSe_2_ as an acceptor, enhancing the adsorption process. This report demonstrated that the sensing capabilities of the VSe_2_ monolayer can be greatly enhanced by incorporating selenium defects into its lattice structure.

Yang et al. [12] developed a colorimetric sensor using AuNPs for the determination of ochratoxin A. The presence of the positive TAMRA tag enhanced signal detection by attracting and concentrating silver lactate around the AuNPs. This increased the likelihood of Ag reduction and deposition catalyzed by the AuNPs. The authors reported that such a strategy is crucial for long aptamers containing a large number of base pairs. A good detection limit of 28.18 pg/mL was achieved using AuNPs.
